# Efficacy of qualitative response assessment interpretation criteria at 18F-FDG PET-CT for predicting outcome in locally advanced cervical carcinoma treated with chemoradiotherapy

**DOI:** 10.1007/s00259-016-3537-8

**Published:** 2016-10-14

**Authors:** Andrew Scarsbrook, Sriram Vaidyanathan, Fahmid Chowdhury, Sarah Swift, Rachel Cooper, Chirag Patel

**Affiliations:** 1 Department of Radiology, Leeds Teaching Hospitals NHS Trust, Leeds, UK; 2 Department of Nuclear Medicine, Leeds Teaching Hospitals NHS Trust, Level 1, Bexley Wing, St James’s University Hospital, Becket Street, Leeds, LS9 7TF UK; 3 Leeds Institute of Cancer and Pathology, University of Leeds, Leeds, UK; 4 Department of Clinical Oncology, Leeds Teaching Hospitals NHS Trust, Leeds, UK

**Keywords:** FDG PET-CT, cervical cancer, therapy assessment, radiotherapy, survival analysis, qualitative scoring

## Abstract

**Objectives:**

To evaluate the utility of a standardized qualitative scoring system for treatment response assessment at 18F-FDG PET-CT in patients undergoing chemoradiotherapy for locally advanced cervical carcinoma and correlate this with subsequent patient outcome.

**Methods:**

Ninety-six consecutive patients with locally advanced cervical carcinoma treated with radical chemoradiotherapy (CRT) in a single centre between 2011 and 2014 underwent 18F-FDG PET-CT approximately 3 months post-treatment. Tumour metabolic response was assessed qualitatively using a 5-point scale ranging from background level activity only through to progressive metabolic disease. Clinical and radiological (MRI pelvis) follow-up was performed in all patients. Progression-free (PFS) and overall survival (OS) was calculated using the Kaplan-Meier method (Mantel-Cox log-rank) and correlated with qualitative score using Chi-squared test.

**Results:**

Forty patients (41.7 %) demonstrated complete metabolic response (CMR) on post-treatment PET-CT (Score 1/2) with 38 patients (95.0 %) remaining disease free after a minimum follow-up period of 18 months. Twenty-four patients (25.0 %) had indeterminate residual uptake (ID, Score 3) at primary or nodal sites after treatment, of these eight patients (33.3 %) relapsed on follow-up, including all patients with residual nodal uptake (n = 4Eleven11 of 17 patients (64.7 %) with significant residual uptake (partial metabolic response, PMR, Score 4) subsequently relapsed. In 15 patients (15.6 %) PET-CT demonstrated progressive disease (PD, Score 5) following treatment. Kaplan-Meier analysis showed a highly statistically significant difference in PFS and OS between patients with CMR, indeterminate uptake, PMR and PD (Log-rank, P < 0.0001). Chi-squared test demonstrated a highly statistically significant association between increasing qualitative score and risk of recurrence or death (P < 0.001).

**Conclusion:**

Use of a 5-point qualitative scoring system to assess metabolic response to CRT in locally advanced cervical carcinoma predicts survival outcome and this prognostic information may help guide further patient management.

## Introduction

Cervical cancer is the 4th most common malignancy worldwide in females with more than 527,000 new cases diagnosed in 2012 [1]. Radical chemoradiotherapy (CRT) is the standard treatment option for patients with locally advanced disease [2]. Up to a third of patients develop disease recurrence usually within the first 2 years after treatment [3]. Use of fluorine-18 fluorodeoxyglucose (FDG) positron emission tomography-computed tomography (PET-CT) to assess response in the post-therapy setting has been evaluated in a number of prospective single-centre studies and is found to predict independently patient outcome [4–7]. Despite the findings of these studies, widespread use of FDG PET-CT in this setting has yet to enter routine clinical practice and many centres continue to use a combination of clinical examination and magnetic resonance imaging (MRI) to evaluate response to CRT. MRI assessment post-CRT can be challenging with a risk of false-positive results due to residual post treatment changes and repeat imaging is required in this circumstance to avoid unnecessary surgical intervention [8].

FDG PET-CT is now the standard of care for response assessment in FDG-avid lymphoma and classification using an internationally recognized 5-point scale (Deauville criteria) is well established [9]. Similar 5-point qualitative interpretative criteria for FDG PET-CT response assessment post CRT have been reported to have substantial inter-reader agreement and excellent negative predictive value in other clinical scenarios including head and neck cancer [10, 11] and lung carcinoma [12]. Five-point qualitative interpretation criteria have not been formally evaluated for response assessment following CRT in locally advanced cervical cancer (LACC) to the best of our knowledge. The purpose of this study was to evaluate the utility of a standardized qualitative scoring system for response assessment at FDG PET-CT in patients undergoing CRT for LACC in a large-volume tertiary referral centre and correlate this with subsequent patient outcome.

## Materials and methods

### Patient cohort

This was a retrospective study performed under a waiver of informed consent approved by the institutional review board. Ninety-six female patients with histologically confirmed LACC treated with curative-intent CRT at a single institution between February 2011 and April 2014 were included. Consecutive patients who underwent pre-treatment imaging and FDG PET-CT following completion of CRT were identified from an institutional database. Patient characteristics, staging, treatment, and follow-up details were recorded.

### Treatment

During the study period, external beam radiotherapy was delivered to the pelvis to a dose of 48 Grays (Gy) in 24 fractions. Concurrent weekly cisplatin chemotherapy to a dose of 40 mg/m^2^ was administered during the external beam component of radiotherapy. Within 10 days of completion, a high-dose rate intra-cavitary brachytherapy boost was delivered as three fractions over 3 weeks in the majority of patients. In patients with issues preventing intra-cavitary treatment or with poor initial response an external beam boost of 12-18 Gy was given instead.

### PET-CT technique

All FDG PET-CT scans were performed using a Philips Gemini TF 64 scanner (Philips Healthcare, Netherlands). Serum blood glucose was checked routinely and imaging was not performed in patients with a blood glucose level of > 10 mmol/L. Patients fasted for at least 6 h prior to intravenous injection of 400 MBq of 18F-FDG. PET acquisition from skull base to upper thighs was performed 60 min after tracer injection. The CT component was performed according to a standardised protocol (without the use of iodinated contrast medium) with the following settings: 140 kV; 80 mAs; tube rotation time 0.5 s per rotation; pitch 6; section thickness 3.75 mm (to match the PET section thickness). Patients maintained normal shallow respiration during the CT acquisition. Images were reconstructed using a standard OSEM algorithm with CT for attenuation correction. Both non-attenuation corrected and attenuation corrected datasets were reconstructed.

### PET-CT interpretation criteria

**Fig. 1:**
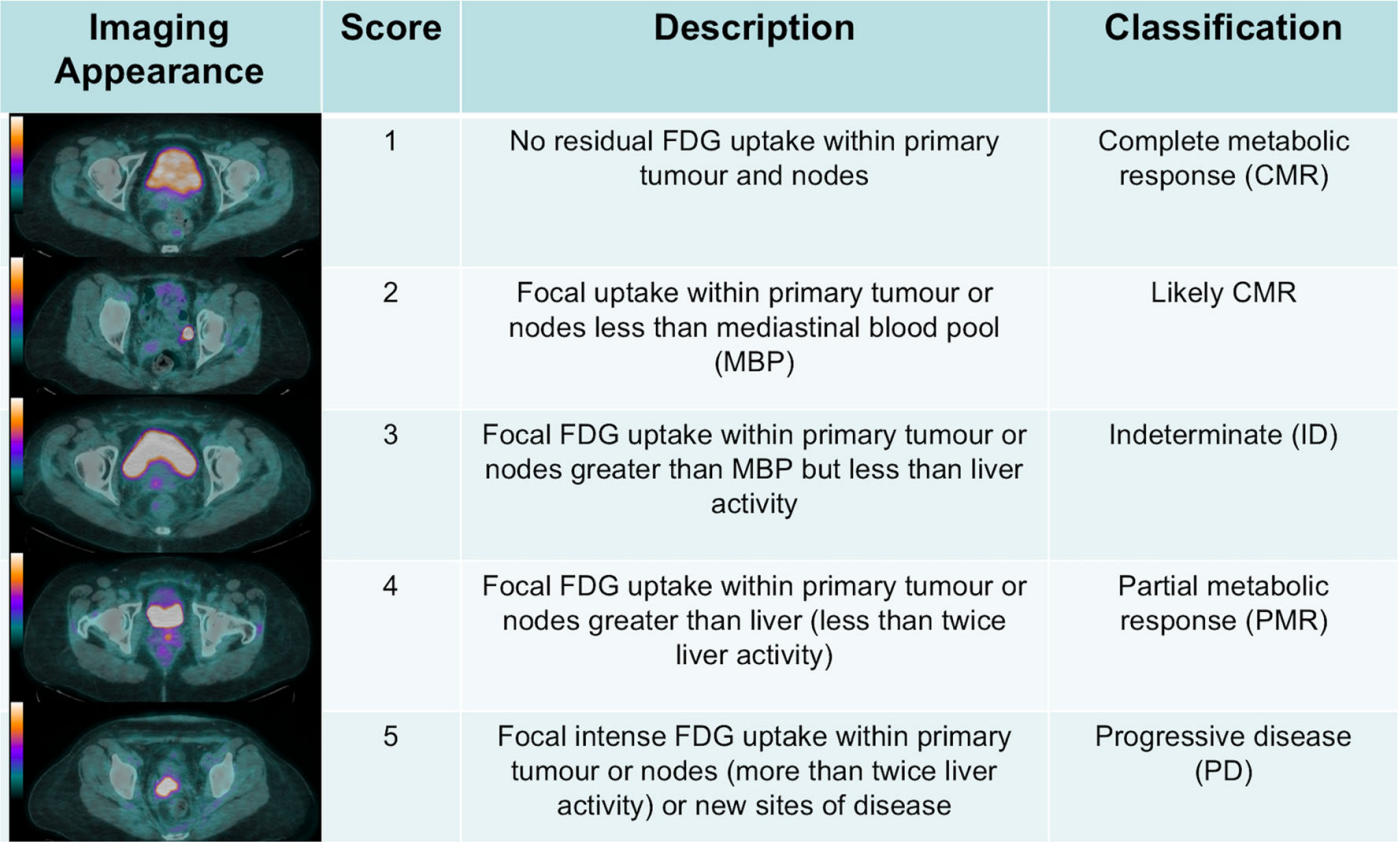
Five-point qualitative response assessment scoring system for locally advanced cervical carcinoma treated with chemoradiotherapy

Metabolic response was assessed qualitatively using a 5-point scale ranging from background level activity only through to progressive metabolic disease (Fig. 1). No residual FDG uptake within primary tumour and nodes compared to surrounding background soft tissue activity was scored as 1, consistent with complete metabolic response (CMR). Focal uptake less than mediastinal blood pool (MBP) activity was scored as 2, consistent with likely CMR. Focal uptake greater than MBP, but less than liver activity was scored as 3 and classified as indeterminate (ID). Focal uptake greater than liver activity was scored as 4, consistent with partial metabolic response (PMR). Focal, intense uptake greater than twice background hepatic activity or new foci not present on baseline imaging were scored as 5, progressive disease (PD). All studies were interpreted by a team of three experienced dual-accredited radiologists and nuclear medicine physicians with a minimum of 7 years’ experience of reporting oncologic PET-CT using specialised software (Advantage Windows Version 4.5, GE Healthcare, Chalfont St. Giles, UK).

### Clinical follow-up

Clinical follow-up of patients with physical examination was performed 6 weeks following completion of CRT and every 3 months until 2 years post-therapy. MRI scans were performed at 3 and 12 months. An additional MRI was performed at 6 months if the 3-month scan was indeterminate. Recurrence was confirmed histologically or by evidence of progression on sequential imaging.

### Statistical analysis

Times to event were measured from the date of completion of CRT. Progression-free (PFS) and overall survival (OS) was calculated using the Kaplan-Meier method (Mantel-Cox log-rank) and correlated with qualitative score using Chi-squared test. Correlation between time interval from end of treatment to imaging and response assessment score was assessed using Pearson’s correlation coefficient. Statistical analysis was performed using IBM SPSS Statistics for Macintosh (Version 20, IBM Corp, Armonk, NY, USA).

## Results

### Patient characteristics

Ninety-six female patients (mean age 47 years, range 24-75 years) with histologically confirmed LACC treated with curative-intent CRT were included in the study. Patient characteristics are detailed in Table 1.

**Table 1:** Characteristics of the 96 patients studied

Characteristic	Number
Mean age in years (range)	47 (24 -75)
Histology	
* Squamous*	72 (75 %)
* Adenocarcinoma*	19 (19.8 %)
* Adeno*/*Squamous*	3 (3.1 %)
* Neuroendocrine*	2 (2.1 %)
Stage (FIGO^1^)	
* 1B1*	5 (5.2 %)
* 1B2*	7 (7.3 %)
* 2A*	2 (2.1 %)
* 2B*	65 (67.7 %)
* 3A*	1 (1 %)
* 3B*	7 (7.3 %)
* 4A*	9 (9.4 %)
Nodal disease at baseline	
* Yes*	57 (59.4 %)
* No*	39 (40.6 %)
Mean primary tumour SUV_max_ (Range)	14.6 (0 – 41.3)
Treatment Modality	
* EBRT and intra-cavitary brachytherapy*	65 (67.7 %)
* EBRT only* (*with boost*)	31 (32.3 %)
Disease recurrence	
* Yes*	35 (36.5 %)
* No*	61 (63.5 %)

^1^FIGO = International Federation of Gynaecology and Obstetrics

EBRT = External beam radiotherapy

### Time interval of post-therapy imaging and follow-up

**Fig. 2:**
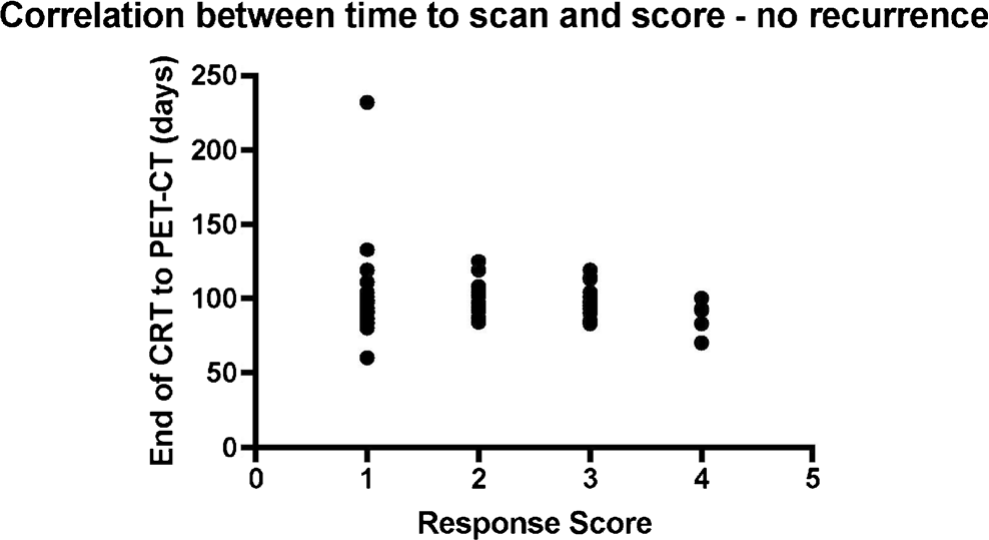
Scatter plot demonstrating the relationship between scanning interval (post-treatment) and response assessment score in patients who did not recur during the follow-up period

All patients had baseline FDG PET-CT pre-treatment and underwent FDG PET-CT approximately 3 months following completion of CRT (mean time elapsed between completion of treatment and response assessment PET-CT was 98.3 days, range 60-232 days). Of the 96 patients in the study 79 (82.3 %) had a scan 3 months +/- 10 days after treatment. Three patients (3.1 %) had a scan less than 80 days after treatment (60, 70, and 78 days, respectively), and 14 patients (14.6 %) had a scan more than 110 days after treatment (of these only one patient had a scan more than 130 days after treatment, at 230 days). These differences in scheduling were largely related to scanner and patient availability. All patients were followed up until death or end of December 2015 (minimum follow-up period of surviving patients 18 months from date of response assessment PET-CT, range 18 – 54 months). There was no statistically significant correlation between time interval from completion of CRT to PET-CT and response assessment score (Pearson’s correlation coefficient, P = 0.32) for patients who did not subsequently recur (Fig. 2), i.e. there was no clear cut-off time after which incidence of indeterminate FDG uptake not due to residual tumour diminished.

### Classification of PET-CT studies

**Fig. 3:**
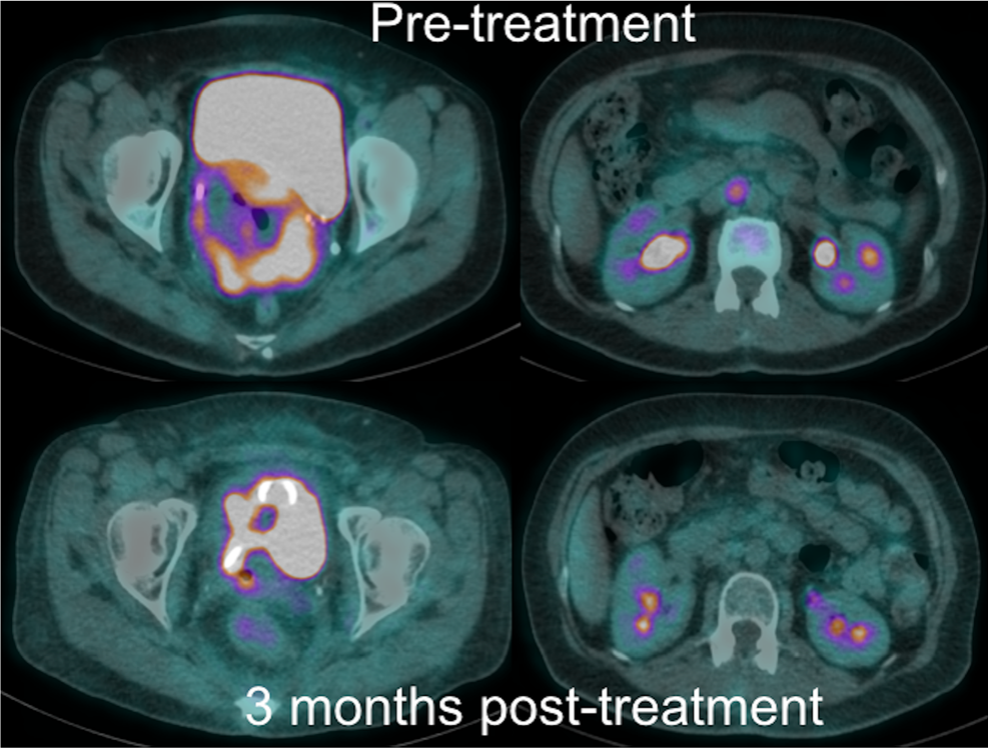
PET-CT imaging performed at baseline (top row) and 3 months post-CRT (bottom row) in a 46-year-old patient with stage 4A disease shows a bulky primary tumour with distal right ureteric involvement (top left) and isolated aortocaval nodal disease (top right). Following treatment there was a good metabolic and morphological response to treatment with only a small residual indeterminate focus of tracer uptake within the cervix (Score 3, bottom left) and minimal activity within the aortocaval node (Score 2, bottom right). The patient relapsed 9 months post-treatment with the development of extensive nodal disease, liver and lung metastases (not shown). She was treated with palliative chemotherapy

**Fig. 4:**
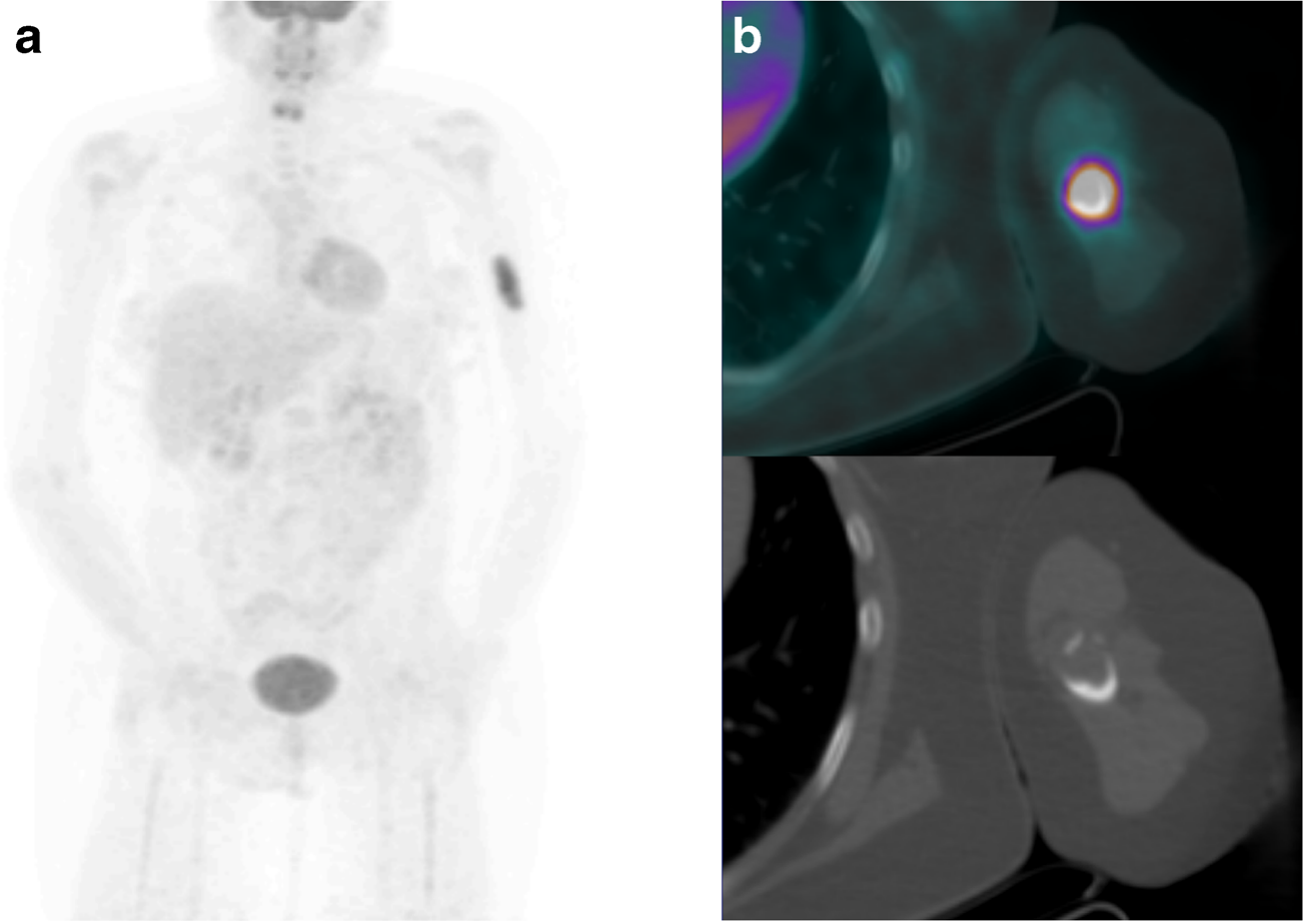
PET-CT imaging performed 3 months post-CRT in a 40-year-old patient with stage 2B disease demonstrates CMR in the pelvis, but a new left humeral bone metastasis (Score 5, PD). This was treated with radiotherapy, but the patient had progressive metastatic disease elsewhere and died 18 months post-initial diagnosis

Of the 96 patients enrolled in the study 40 (41.7 %) demonstrated CMR on post-treatment PET-CT (Score 1 or 2) with 38 patients (95.0 %) remaining disease free after a minimum follow-up period of 18 months. Twenty-four patients (25.0 %) had indeterminate residual primary or nodal uptake (Score 3) after treatment, of which eight patients (33.3 %) relapsed at follow-up (Fig. 3), including all patients with residual nodal uptake (n = 4). Seventeen patients (17.7 %) had significant residual uptake (Score 4), of which 11 (64.7 %) relapsed. Fifteen patients (15.6 %) demonstrated progressive disease (Score 5) following treatment on the response-assessment PET-CT (Fig. 4), of which only one (6.7 %) turned out subsequently to have been falsely positive. Table 2 details disease response classification by treatment modality and outcome.

**Table 2:** Response classification by treatment modality and outcome for the 96 patients

Treatment Modality/Outcome	Number
EBRT and intra-cavitary brachytherapy (No recurrence)	51
*CMR* (*Score 1 or 2*)	33 (64.7 %)
*ID* (*Score 3*)	12 (23.5 %)
*PMR* (*Score 4*)	5 (9.8 %)
*PMD* (*Score 5*)	1 (2.0 %)
EBRT and intra-cavitary brachytherapy (Recurrence)	14
*CMR* (*Score 1 or 2*)	1 (7.1 %)
*ID* (*Score 3*)	4 (28.6 %)
*PMR* (*Score 4*)	3 (21.4 %)
*PMD* (*Score 5*)	6 (42.9 %)
EBRT only (No recurrence)	10
*CMR* (*Score 1 or 2*)	5 (50 %)
*ID* (*Score 3*)	4 (40 %)
*PMR* (*Score 4*)	1 (10 %)
*PMD* (*Score 5*)	0
EBRT only (Recurrence)	21
*CMR* (*Score 1 or 2*)	1 (4.8 %)
*ID* (*Score 3*)	4 (19.0 %)
*PMR* (*Score 4*)	8 (38.1 %)
*PMD* (*Score 5*)	8 (38.1 %)

EBRT = External Beam Radiotherapy

CMR = Complete Metabolic Response

ID = Indeterminate

PMR = Partial Metabolic Response

PMD = Progressive Metabolic Disease

### Overall performance of PET-CT

The overall sensitivity and specificity of PET-CT in predicting residual or recurrent disease was 94.3 % (95 % confidence interval, CI 80.8 – 99.3 %) and 62.3 % (95 % CI 49.0 – 74.4 %), respectively. The positive predictive value was 58.9 % (95 % CI 45.0 – 71.9 %), and the negative predictive value was 95.0 % (95 % CI 83.1 – 99.4 %). The overall diagnostic accuracy in this patient cohort was 74 %.

### Survival outcomes

Thirty-five patients (36.5 %) developed disease progression during the follow-up period between the time of the response assessment PET-CT and death or last patient encounter at our institution; 21 patients in this group (60 %) did not receive intra-cavitary brachytherapy. The mean time to recurrence was 7.3 months (range 2.6 – 38 months). Eight of 24 patients with indeterminate response (Score 3) on post treatment PET-CT subsequently recurred, of which four were successfully salvaged (two had pelvic exenteration for local relapse, and two had CRT for localized nodal relapse). The other four patients were treated with palliative chemotherapy.

Eleven of 17 patients with partial metabolic response (Score 4) had disease recurrence, of which two were successfully salvaged with pelvic exenteration. The other nine patients were treated with palliative chemotherapy.

**Fig. 5:**
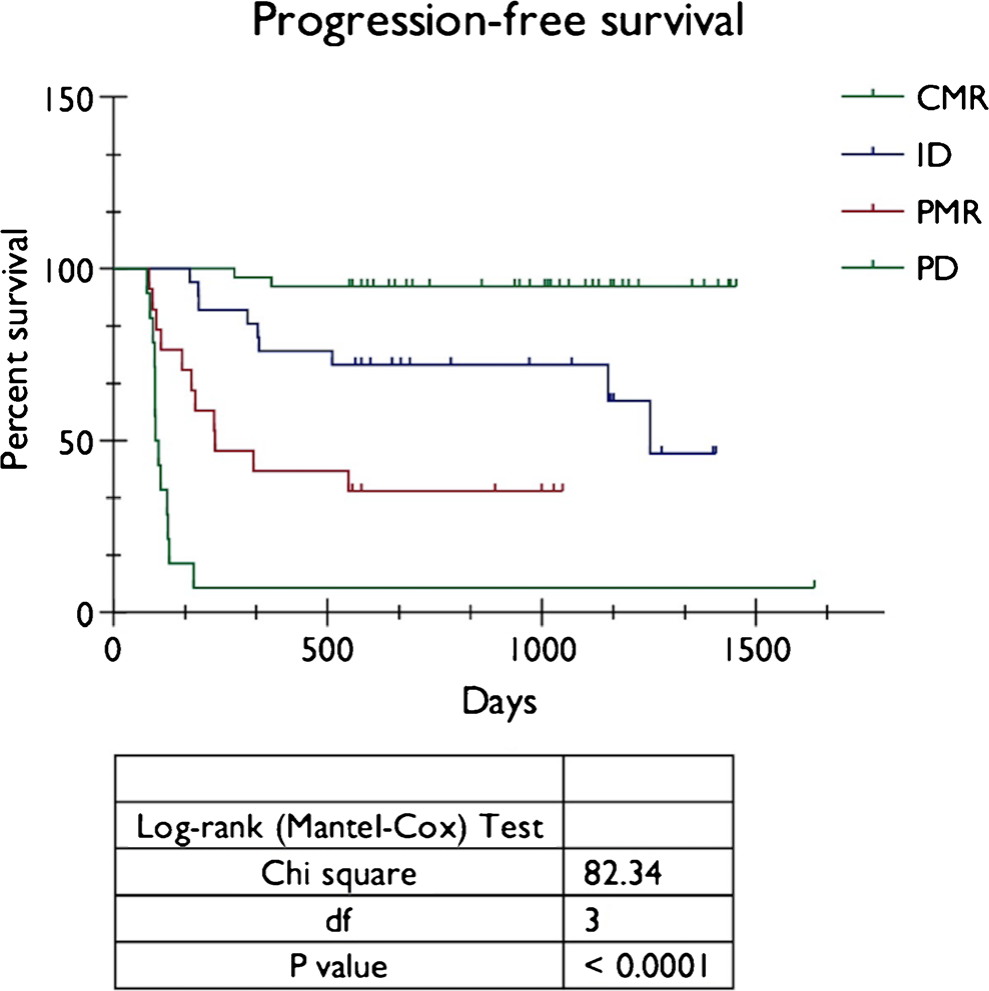
Kaplan-Meier analysis of progression-free survival (PFS) in relation to metabolic response on post treatment PET-CT (CMR = Score 1 and 2, ID = Score 3, PMR = Score 4, PD = Score 5)

**Fig. 6:**
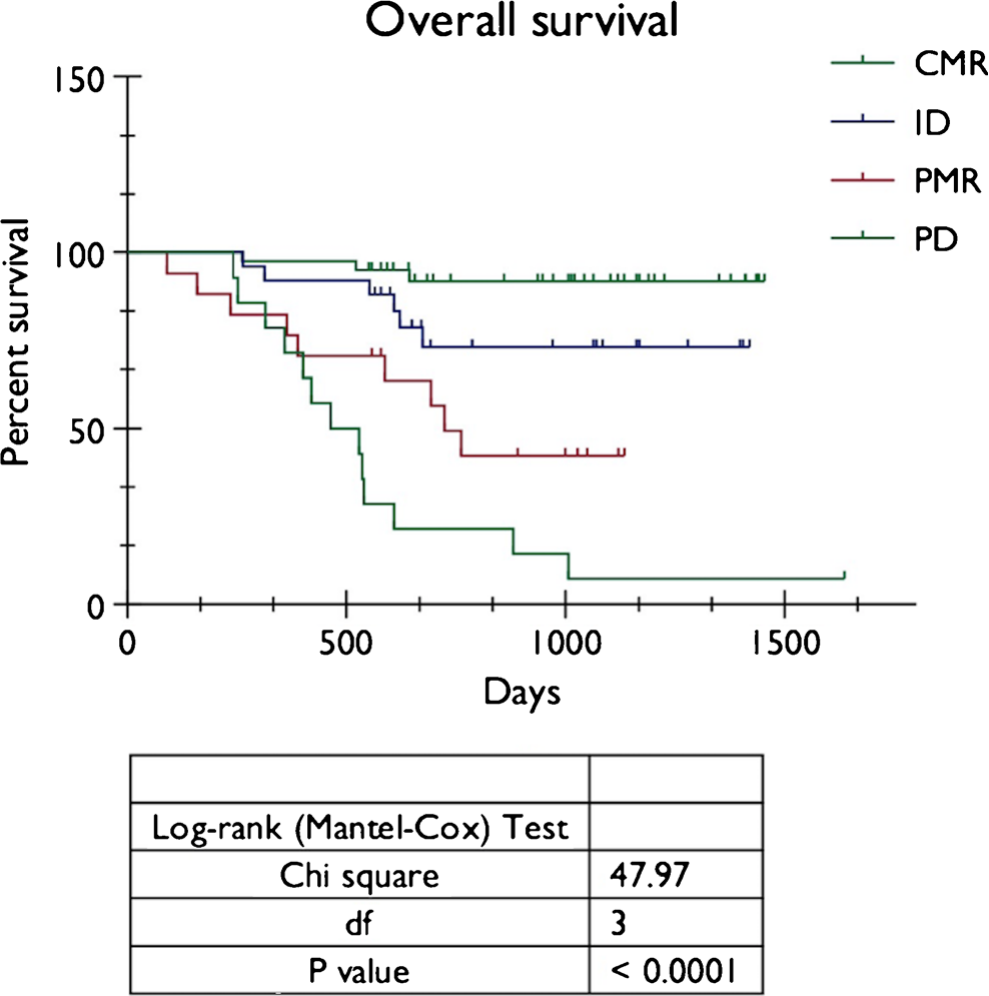
Kaplan-Meier analysis of overall survival (OS) in relation to metabolic response on post treatment PET-CT (CMR = Score 1 and 2, ID = Score 3, PMR = Score 4, PD = Score 5)

Twenty-seven patients died during the follow-up period, of which only one was a non-disease related death (incidental thromboembolic disease). The average time to death was 16.9 months (range 7.8 – 33.1 months). Kaplan-Meier analysis showed a highly statistically significant difference in PFS (Fig. 5) and OS (Fig. 6) between patients with Score 1 or 2 (CMR), Score 3 (ID), Score 4 (PMR), and Score 5 (PD) (Long-rank, P < 0.0001). Chi-squared test demonstrated a highly statistically significant associated between increasing qualitative score and risk of recurrence or death (P < 0.001).

## Discussion

Use of FDG PET-CT to assess post-CRT response in LACC has been evaluated in a number of prospective single centre studies and found independently to predict patient outcome [4–7]. Additional key roles of FDG PET-CT are to flag patients with loco-regional treatment failure potentially suitable for salvage therapy and to identify patients who have developed unsuspected asymptomatic distant metastatic disease not detected on clinical examination. Despite these data, post-therapy PET-CT is not yet routinely recommended in LACC patients who have undergone CRT [13].

The largest prospective study evaluated 238 patients with LACC and reported a 23 % relapse rate in patients with CMR [7]. The relapse rate in CMR patients in other studies ranged from 10-21 % [5, 6] compared to 5 % in our patient cohort. Previous studies have not used a standardized qualitative scoring system and have performed PET-CT at varying time points post-treatment, which may in part explain this difference. One of the groups have recently reported long-term 5-year survival outcomes in their prospective patient cohort and confirmed that CMR on post-treatment PET-CT remains a powerful predictor of survival with a 97 % 5-year overall and 86 % progression-free survival rate [14]. Few published studies have reported the diagnostic accuracy of PET-CT in this clinical scenario. The NPV in our series (95 %) is comparable to that reported (97 %) in a large series of 276 cervical cancer patients undergoing post-treatment PET-CT although the median time between treatment and post-therapy scan was 24 months in their study [15].

A potential issue with the use of FDG PET-CT post-CRT is the lack of specificity of tracer uptake as evidenced by the low specificity (62.3 %) in our series. In particular, there is a potential for false-positive uptake in some patients, with partial metabolic response as a result of persistent post radiation inflammation. The largest study of LACC patients reported that only 26 patients (65 %) with PMR on response-assessment PET-CT had biopsy proven residual disease [7]. In our study the degree of residual uptake in the primary tumour was associated with an increasing risk of relapse: however, a significant number of patients with Score 3 (16 patients, 67.7 %) and Score 4 (six patients, 35.3 %) did not relapse during the follow-up period, which explains the sub-optimal PPV (61.1 %). Interestingly, all patients with residual nodal uptake greater than background liver activity went on to relapse in our study. There was no statistically significant correlation between the time interval from end of treatment to PET-CT imaging and response assessment score in patients who did not subsequently recur. The optimal timing of post-treatment PET-CT response assessment remains uncertain with one group advocating a longer delay of 6 months before imaging to reduce the incidence of false positive inflammatory activity albeit with a small risk of missing early asymptomatic recurrence [5, 14]. A number of different groups have employed a similar time interval to ourselves ranging between 8 and 16 weeks [4, 6–16]. More recently, a number of small studies have evaluated the use of early response assessment with PET-CT either during or within a month of completing CRT to predict relapse in LACC patients with promising results, but no clear consensus [17–19]. The high negative predictive value in the current study (95 %) suggests 3 months may be an acceptable time point when combined with objective assessment although this should be confirmed in a larger prospective multi-centre trial.

How best to manage equivocal or indeterminate response remains unclear and requires further study. Several groups have reported the use of early repeat PET-CT imaging after an initial indeterminate result as an alternative to salvage surgery in head and neck cancer patients [20–23], but the safety of this strategy has not been reported in LACC. Since the majority of LACC patients routinely undergo MRI post-treatment, a combined standardized MRI and PET-CT response assessment scoring system might help increase specificity particularly if this were to encompass diffusion weighted MRI where an increase in apparent diffusion coefficient following treatment has been reported to correlate well with complete response [24, 25]. This warrants further evaluation in a future study.

There was a lower incidence of disease relapse in patients who received high dose rate intra-cavitary brachytherapy (14 of 61 patients, 23.0 %) compared to those who had EBRT boost (21 of 31 patients, 67.7 %). This is not surprising since the latter group generally had more advanced disease, the radiation dose received without brachytherapy is generally lower and it is well recognised that treatment without brachytherapy is associated with lower survival rates [26, 27]. Despite the relatively low PPV in this series, there was clear evidence of persisting disease or disease progression on the post treatment scan in 15 patients (15.6 %) which provided additional information and helped guide appropriate further management. One patient in this group had new pelvic nodal uptake interpreted as disease progression which subsequently resolved spontaneously with no evidence of disease relapse during the follow-up period. This is likely to have been due to treatment related inflammation.

A recent large study of 362 patients with head and neck cancer reported the safety and cost-effectiveness of a less intensive clinical follow-up strategy in patients with CMR on a 3-month post-treatment FDG PET-CT with reduction in frequency of follow-up from 3 to 6 months with no apparent clinical detriment and reduced costs [28]. A similar study in patients with LACC has not been reported and this warrants further evaluation to assess the safety and validity of risk-adapted follow-up based on post-treatment PET-CT response.

There are a number of limitations to this study, including the single institution and retrospective nature, relatively small cohort size, and not all patients received the same treatment, with some patients not suitable for high-dose brachytherapy. There is growing evidence of the superior utility of objective 5-point scoring systems for PET-CT assessment of treatment response in a variety of tumour types [9–11]. Despite the stated limitations, the potential superior efficacy of a standardized 5-point scoring system for response assessment PET-CT in patients with LACC has been demonstrated. This requires validation in a larger prospective multi-centre study, which could be designed to guide a less invasive and resource-intensive follow-up strategy for patients demonstrating CMR.

## Conclusions

Use of a 5-point qualitative scoring system to assess metabolic response to CRT in locally advanced cervical carcinoma predicts survival outcome and this prognostic information may help guide further patient management.
